# Advances in Duplex Stainless Steels

**DOI:** 10.3390/ma15207132

**Published:** 2022-10-13

**Authors:** Luca Pezzato, Irene Calliari

**Affiliations:** Department of Industrial Engineering, University of Padova, Via Marzolo 9, 35131 Padova, Italy

Duplex stainless steels (DSSs) are a group of stainless steels characterized by a biphasic microstructure consisting of ferrite and austenite. They are widely used in hostile environments, such as nuclear and petrochemical plants, oil and gas offshore applications, chemical plants, the paper and pulp industry, and the food and beverages industry as an alternative to the austenitic stainless steels. The optimization of the properties of duplex stainless steels can be achieved through a proper balance of ferrite and austenite. This balanced ratio between the two phases (50% of ferrite and 50% of austenite) is obtained with a suitable combination of chemical composition and solution heat treatment.

However, due to the presence of the metastable austenitic phase and the instability of ferrite at high temperatures, these steels are sensitive to diffusive and diffusionless phase transformations, which may affect corrosion and mechanical properties, but there is a lack of research in the field of physical metallurgy regarding these transformations.

Despite their diffusion as consolidated materials, many research fields are also focusing on the possibility of increasing duplex mechanical properties and corrosion resistance by composition and heat treatment optimization. Furthermore, special innovations are soon to be realized in the field of welding processes.

This Special Issue presents a selection of interdisciplinary studies that cover physical metallurgy and processes and report on the experimental and theoretical progress concerning microstructural evolution during processing, microstructure–properties relations, and applications. Moreover, two papers regarding innovations in the welding of DSS_s_ are also presented in the issue. In this editorial, the editors wish to briefly introduce the different articles published in the Special Issue. 

Gennari et al. [1] studied the influence of electropulsing treatment on pre-strained UNS S32750 duplex stainless steel. They found that electropulsing treatments conducted on 5% and 15% prestrained specimens almost eliminate the work-hardening state in the first case, while partially recovering the work-hardened state in the second. The effect was caused by a combined effect of increase of atomic flux, due to the electrical current and by local joule heating in correspondence with crystal defects. Consequently, electropulsing treatment applied to metallic alloys was identified as a promising technique to reduce the work-hardening state without the need of annealing treatments in a dedicated furnace.

Rodriguez et al. [2] investigated the influence of the parameters employed in the gas tungsten arc welding process (GTAW), where nickel powder is used as a filler metal in 2304/2507 duplex stainless-steel dissimilar joints. They found that a nickel powder addition combined with a low heat input increased the biphasic ratio across the different zones of the dissimilar welded samples. Although the austenite volume fraction increased to 25% in the 2304 heat-affected zone (HAZ), it was not sufficient according to international standards. The obtained results led to the maintenance of the 50/50 phase percentage in the 2507 HAZ welded joint side, as well as an increment of the austenite percentage in the 2304 HAZ. The finding obtained in this study can be employed by welding engineers to improve the austenite/ferrite balance with nickel powder addition in the duplex stainless-steel dissimilar joints. 

Biserova-Tahchieva et al. [3] focused their study on the effect of a plasma ion carburizing process on duplex and superduplex stainless steels (DSS and SDSS), in order to analyze the influence of diffusion elements on the precipitation of secondary phases after additional short thermal treatment. The precipitation of secondary phases was observed at a temperature of 925 °C during the large period and after normal cooling. A sigma phase after the plasma cycle was commonly identified in both duplex and superduplex samples. On the other hand, a chi-phase in the SDSS was found in the inner microstructure. The results obtained by these authors show that a plasma-carburizing procedure at a relatively high temperature is an effective method for protection against surface corrosion. In fact, corrosion potentials were almost equal for both DSS and SDSS samples, and although precipitation occurred in the interior, the surface of the sample, being thermochemically protected, was not altered.

Silva Leite et al. [4] investigated the Nd: YAG pulsed laser dissimilar welding of UNS S32750 super duplex stainless steel (SDSS) with 316L austenitic stainless steel (ASS), using different heat inputs. They found that the fusion zone microstructure consisted of a ferrite matrix with grain boundary austenite (GBA), Widmanstätten austenite (WA) and intragranular austenite (IA), with the same proportion of ferrite and austenite phases. Changes in the heat input (between 45, 90 and 120 J/mm) did not significantly affect the ferrite/austenite phase balance and the microhardness in the fusion zone. Considering the obtained results, they conclude that the Nd: YAG pulsed laser welding of dissimilar materials—namely, UNS S32750 super duplex stainless steel (SDSS) with AISI 316L austenitic stainless steel (ASS)—is recommended.

Barella et al. [5] presented a study related to low-density steels, and their analysis concerned the corrosion and hot oxidation resistance of a Fe-15%Mn-9.5%Al-6.5%Ni-1%Cr-0.43%C. Material behavior was analyzed in both as-cast and heat-treated states. The corrosion tests showed a good corrosion resistance for the as-cast material, comparable to austenitic stainless steels at low temperatures, and an excellent resistance of the 1300 °C solution-treated samples, especially at low temperatures but also at high temperatures. In detail a corrosion resistance behavior comparable to the AISI 304 austenitic stainless steel was pointed out. The authors concluded that the results obtained for this Fe-15%Mn-9.5%Al-6.5%Ni-1%Cr-0.43%C alloy are promising for the exploitation of this steel grade in those applications where corrosion resistance in chlorine-rich environments or hot oxidation resistance are relevant.

Mészáros et al. [6] investigated the eutectoidal phase transformation of 2507-type super-duplex stainless steel. The results demonstrate the decomposition of the δ-ferrite into the σ-phase and secondary austenite with the same tendency. It was clearly demonstrated that previous cold rolling before heat treatment significantly increases the rate of eutectoidal decomposition and decreases its starting temperature. The activation energy of the δ-ferrite decomposition process was also determined. The obtained activation energy for the undeformed sample was 302 kJ/mol, and its value decreased due to previous cold rolling to 296 kJ/mol. These values are close to the activation energy values of the diffusion process of chromium and molybdenum in ferrite. Therefore, the authors concluded that the rate-limiting step of the whole diffusion-controlled phase transformation of δ-ferrite is the diffusion of these two alloying elements.

Gennari et al. [7] studied the effect of electrical current and joule heating on the deformation of metals, known as the electroplastic effect (EPE), on different grades of duplex stainless steels. Tensile tests at 5 A/mm^2^, 10 A/mm^2^ and 15 A/mm^2^ current densities, along with their thermal counterparts, were conducted on UNS S32101, UNS S32205, UNS S32304 and UNS S32750. An increase in uniform elongation for the electrical tests compared to their thermal counterparts, as well as an increase in total elongation, was found. No differences were observed in the yield stress and ultimate tensile strength. An uneven distribution of the current because of the different resistivity and work hardening of the two phases has been hypothesized as the explanation for the positive effect of EPE. The authors concluded that EPE can be exploited in the manufacturing of DSS, such as in wire drawing.

Generally, all the articles presented in the Special Issue are derived from collaborations between different institutions across the world and this, in the editors’ opinion, plays a crucial role in the high quality of the performed research. Collaboration is vital to ensure that innovative research is conducted in the future.

In conclusion, the editors wish to give a special thanks to all the authors and the editorial team of *Materials* for the collaborative peer-review process. As for the readers, we hope you enjoy reading this Special Issue “Advances in Duplex Stainless Steels” and discover new innovations to inspire future research.

## Data Availability

Not applicable.
